# Book Notices

**Published:** 1899-11

**Authors:** 


### BOOK NOTICES.

REPERTORY OF THE URINARY ORGANS AND PROSTATE GLAND, INCLUDING CONDYLOMATA. Compiled by A. R. Morgan, M. D. Philadelphia: Bœricke & Tafel, 1899. Price, half morocco, $3.00 net; by mail, $3.10.

This most excellent, practical, and soundly homœopathic book, is warmly welcomed to our editorial table.

In his preface the author says, "The work might undoubtedly have been better." This *is* doubtful. The work could *not* have been better. Then he goes on to say, "but it certainly could not have been more conscientiously done." This we unreservedly believe. Its carefulness and its minute elucidation of the symptomatology entitle it to all praise, all confidence of the practical prescriber seeking to find the true simillimum with the invincible determination to cure his case.

Its division and sub-division are somewhat peculiar. But it can be readily learned. We note some of these divisions: Kidney, Ureters, Bladder, Urethra, Meatus Urinarius, Fossa Navicularis, Desire to Urinate, Emission of Urine before Micturition. On Beginning Micturition, At Close of Micturition, After Micturition, Before and During Micturition, Before and After Micturition, During and After Micturition, Before, During, and After Micturition, Between Acts of Micturition, Character of Urine, Color of Urine, and so on. A glance at these headings will convince the homœopathist of the pains-taking character of the preparation of this book. Sediments and odors are given and the remedies are indicated in three kinds of type, according to their importance, after the manner of Bœnninghausen. Further on we find a separate repertory of the prostate gland, and then come some diagnostic tables, which are excellent, and the several urinary tests.

The diagnostic table is especially commendable as it enables us to interpret the meaning of any symptom or appearance of the urine, at a glance. We observe one slight omission in this table, and that is headache as a frequent cause of temporary increased quantity of urine. This, however, is of no importance, and is utterly insignificant in the presence of the great number of valuable qualities possessed by the work.

It must become at once the every-day companion of the homœopathist and he will not be induced to part with it.

We are happy to note that there is in these days a great increase in the number of practical working books of the Hahnemannian prescribers, and this valuable production must stand out as one of the most distinguished of them because it is instantly, continuously, and readily available in getting the right remedy in prevalent complaints.

The zeal of the author for true Homœopathy is well known. His energetic championship of the cause has even led him at times to severe criticism of his friends.

The editor recalls how at one time, at a public meeting of a society the author of the book under review brought the editor before the society for censure for merely publishing in the pages of this journal the foibles of a professional brother; even going so far as to publish a private letter relating to the subject. For this we bear him no malice notwithstanding we know it was a mistake, but accepted it as being an over-exertion of his energy for the cause and not an exhibition of personal malice.

Bee Line Therapia and Repertory. By Stacy Jones, M. D. Second edition. Philadelphia: Bœricke & Tafel, 1011 Arch Street, 1899. Price, in flexible morocco, $2.00 net; by mail, $2.06.

This is a familar hand-book to most homœopathic physicans now enlarged in the second edition to twice its original size.

It is in alphabetical order so that there is no method of arrangement to be learned in order to use it. Thus we find such words as Aconite Adenitis, After birth, After Pains, Agalactic, Aggravation, showing that its arrangement is that of the dictionary. In other letters of the alphabet we find the same arrangement: names of remedies, particular symptoms, and names of diseases, all following each other as indicated by their first and second letters.

Looking at the word aggravation, we find the following sub-divisions: As to food, worse. Under this heading we find different kinds of food given with the remedies most useful. As to drink, worse; here we find a similar arrangement. As to sound, worse; As to odor, worse; As to weather, worse; As to time, worse; As to heat, worse; As to cold, worse; As to rest, worse; As to motion, worse; as to touch, worse. In these sub-divisions varieties are given and the indicated remedies.

Under Ichthyol, we find the following: "Dose, adult, 2 gr. pill, two or three times a day. For any pain or for any blood or skin disease." We regret to meet with such a statement as this; as, in its attempts to make Homœopathy easy, it reduces it to a practice of empiricism and rule of thrumb, to the injury of the patient and the discredit of the cause. There are several other objectionable things to be noticed about this little book, and consequently we are not able to recommend it to the profession.

Renal Therapeutics, including also the Study of the Etiology, Pathology, Diagnosis and Medical Treatment of Diseases of the Urinary Tract. By Clifford

Mitchell, A. M., M. D. Philadelphia: Bœricke & Tafel, 1898. Price, cloth, $2.00; by mail, $2.16.

Professor Mitchell, the author of this book is well known for his long connection with the faculty of the Chicago Homœopathic College. He has produced a learned work in all that pertains to anatomy, physiology, and pathology. But when he comes to therapeutics his book is lamentably deficient; the treatment being less a matter of Homœopathy than of empiricism.

The cuts illustrating the minute anatomy of the kidney are particularly good and must help the reader to a clear mental picture of the details of the kidney and to an understanding of the phenomena of secreting urine. We regret that we cannot give unqualified praise of its methods of treatment.

The Logic of Figures; or, Comparative Results of Homœopathic and other Treatments. Edited by Thomas Lindsley Bradford, M. D. Philadelphia: Bœricke & Tafel, 1899. Price, cloth, $1.25 net; by mail, $1.32.

As is said in the preface, "This book is a compilation from all available sources of the comparative results of homœopathic and other kinds of medical treatment, both in public institutions and in private practice."

The book represents an immense amount of research conducted by the author in his well-known, thorough way. Yet he says that it is not exhaustive. If not the figures given are of the greatest importance to homœopathists.

The bibliography of statistical books is given so that the reader who may desire to do so can delve into the figures and go deeper, if he wish, than Dr. Bradford himself has gone.

In our opinion what is here given is all that even advanced students of Homœopathy will need for general use, and those who are not students will be greatly impressed with the comparative values given, representing in nearly all cases the superiority of our school of treatment.

Transactions of the Homœopathic Medical Society of the State of New York for the Year 1898. Volume XXXIII. Edited by the Secretary, John L. Moffat, B. S., M. D., O. *et* A. Ch., Brooklyn, New York, 1898.

It is a moral impossibility to do justice to this book within the limits of a review. There are too many subjects and they are too ably treated to leave anything for us to say. Thus if we take the address of the president, Eugene H. Porter, we find that he touches upon a large number of highly important subjects from a public point of view. Thus he opens with a discussion of the question of expert testimony in the courts and its evils. Next he treats of the abuses of medical charity, then upon public medicine, then closes with a definition of what constitutes a homœopathic physician.

The society voted to print the address as a separate pamphlet and it is likely a copy may be obtained of the secretary, Dr. John L. Moffat, 17 Schermerhorn St., Brooklyn, N. Y.

The other papers we have not the time or space to notice.

### On the Relations of Antitoxin Treatment to Homœopathy, including a New Explanation of the Law of Similia. By Emanuel M. Baruch, Ph. D., M. D. New York: Bœricke & Runyon Co., 1899.

This little volume sets forth the success of antitoxin as an indisputable fact in curing diphtheria and calls it a triumph of modern medicine. It then proceeds to claim that its action is homœopathic to diphtheria, and a further proof of the truth of Homœopathy. We are not able to agree with the author in his views and conclusions; but it would take too much time and space to set this forth adequately. So we will simply say we disapprove of antitoxin and consequently disapprove of this book.

### A Text-Book of Materia Medica, and Therapeutics of Rare Homœopathic Remedies. A Supplement to Dr. A. C. Cowperthwaite's *Materia Medica*, or every greater *Materia Medica*. By Oscar Hansen, M. D., Copenhagen, Denmark. London: The Homœopathic Publishing Company, 12 Warwick Lane, Paternoster Row, E. C., 1899. Price, 4 shillings net.

This little book contains all, or nearly all, the new remedies of the materia medica which have come to the notice of the profession since the great books of materia medica, such as the *Encyclopædia* and the *Guiding Symptoms*, have been published.

It is noticeable that these newer remedies are very imperfectly proved. None of them can show the pathogenesis which can be found under Phosphorus, Sulphur, Aconite, Belladonna, and other remedies, which are the masterpieces of the homœopathic healing art, and have made it such an overwhelming success in practice. In this little book these partial provings are still further abbreviated and so we have a small volume of about 120 pages which contains notices of over three hundred and fifty new medicines.

In his desire for abbreviation the author does not state what these new medicines are, and so we are left in a state of ignorance concerning many of them. The work is furnished with an excellent index and a list of authorities from which the data are taken, and thus it forms a ready reference book which has cost its author a large outlay of labor, since he must have gone over almost all literature published in the homœopathic school.

THE PHYSICIAN'S VISITING LIST FOR 1900. P. Blakiston's Son & Co., 1012 Walnut St., Philadelphia.

This visiting list is highly recommended by the editor of this journal who has used none other for twenty years. It is convenient in all respects and being uniform from year to year, the physician becomes familiar with its arrangement and knows just where to look for any memorandum he wants. For sizes and prices the reader is referred to the advertisement.

